# Context-dependent multimodal behaviour in a coral reef fish

**DOI:** 10.1098/rsos.240151

**Published:** 2024-05-01

**Authors:** Isla Keesje Davidson, Ben Williams, John E. Stratford, Lucille Chapuis, Stephen D. Simpson, Andrew N. Radford

**Affiliations:** ^1^ School of Biological Sciences, University of Bristol, Bristol, UK; ^2^ Centre for Biodiversity and Environment Research, University College London, London, UK; ^3^ Leigh Marine Laboratory, Institute of Marine Science, University of Auckland, Auckland, New Zealand

**Keywords:** anti-predator behaviour, behavioural flexibility, signalling, territory defence, trade-off, vocalizations

## Abstract

Animals are expected to respond flexibly to changing circumstances, with multimodal signalling providing potential plasticity in social interactions. While numerous studies have documented context-dependent behavioural trade-offs in terrestrial species, far less work has considered such decision-making in fish, especially in natural conditions. Coral reef ecosystems host 25% of all known marine species, making them hotbeds of competition and predation. We conducted experiments with wild Ambon damselfish (*Pomacentrus amboinensis*) to investigate context-dependent responses to a conspecific intruder; specifically, how nest defence is influenced by an elevated predation risk. We found that nest-defending male Ambon damselfish responded aggressively to a conspecific intruder, spending less time sheltering and more time interacting, as well as signalling both visually and acoustically. In the presence of a model predator compared to a model herbivore, males spent less time interacting with the intruder, with a tendency towards reduced investment in visual displays compensated for by an increase in acoustic signalling instead. We therefore provide ecologically valid evidence that the context experienced by an individual can affect its behavioural responses and multimodal displays towards conspecific threats.

## 1. Introduction

Trade-offs lie at the heart of animal behaviour, with individuals dynamically balancing the risk and reward of different choices to optimize their survival and reproductive success [1–3]. Trade-offs can occur between different behaviours, such as between foraging and vigilance or territory defence [4,5]. Individuals can also face trade-offs related to a single behaviour, such as when choosing between different modes of foraging [6]. In many species, individuals use signals in more than one sensory modality to communicate [7–11]. Multimodal components may signal different information [12] but can also provide the same information and thus be used flexibly depending on circumstances [13,14]; the latter represents a within-behaviour trade-off.

Behavioural decisions are often context-dependent, with flexibility exhibited in relation to, for instance, satiation level, social factors (e.g. the presence of an audience) and environmental conditions, including habitat type and predation risk [15–18]. For example, an increase in predation threat can lead to reductions in foraging, social bonding or territory defence [1,17,18]. In terms of multimodal signalling, animals can preferentially use particular modalities if others will be disrupted by natural or anthropogenic disturbances [13,19,20] or if switching would reduce the threat of predation [11]. Much research on context-dependent behavioural trade-offs has been on terrestrial species, but fish must make such decisions too. For instance, parrotfish (Scaridae) and surgeonfish (Acanthuridae) shift their prioritization of behaviours, such as foraging or predator avoidance, depending on the time of day and the predator type to which they are exposed [21]. In addition, laboratory-housed Lusitanian toadfish (*Halobatrachus didactylus*) altered their aggressive visual displays and defensive acoustic signals under changing social contexts, demonstrating multimodal flexibility [10]. However, this capacity for individuals to shift between sensory modalities has rarely been documented in wild aquatic systems [19].

We used field experiments with Ambon damselfish (*Pomacentrus amboinensis*) to investigate context-dependent behavioural trade-offs, including the use of multimodal signals. Male Ambon damselfish defend their nest against conspecifics using displays with both visual (e.g. fanning of the caudal fins) and acoustic (e.g. production of high-pitched tonal sounds) components [22–24]. We explored how the presence of a predator of adult damselfish affects male defensive actions towards a conspecific intruder, investigating contextual variation in multimodal behavioural responses. We predicted that the presence of a predator would lead to males reducing those behaviours directed at conspecific intruders that require them to be away from shelter and thus exposed to a greater risk. We also predicted a change in the use of multimodal defensive displays, such that the vulnerability of the signaller to predation would be reduced.

## 2. Material and methods

We conducted the research in September–December 2019 at the Lizard Island Research Station (14°40′ S 145°280′ E), Great Barrier Reef, Australia. The study focused on wild Ambon damselfish males defending artificial nests; males defend their nest site throughout the breeding season both when they have eggs to tend and for potential future reproductive opportunities [23,24]. All trials were conducted when there were no eggs in the nests. Experiment 1 established the behavioural responses to a conspecific intruder, including any multimodal component, by comparing two treatments: an intruder presented in a bag versus an empty bag (as a control). Experiment 2 tested how an elevated predation risk affects behavioural responses to a conspecific intruder by comparing two treatments: an intruder in a bag presented at the same time as either a looming predator model or a herbivore model (as a control). We used each focal male for only one experiment (Experiment 1: *n* = 22 and Experiment 2: *n* = 20), each of which had a repeated-measure design. Treatments to a focal fish were presented over two consecutive days, at the same time of day (±2 h); we counterbalanced the treatment order between focal fish. Males used as intruders were caught in different areas from those where focal nests were located. Intruders were measured (mean ± s.d. total length: 7.5 ± 0.8 cm) and visually size-matched to focal individuals; focal individuals were not caught and measured to minimize disruption. We video-recorded trials using GoPro (Hero 7) cameras; see electronic supplementary material for further details on artificial nests, intruder capture and camera set-up.

**Figure 1.**
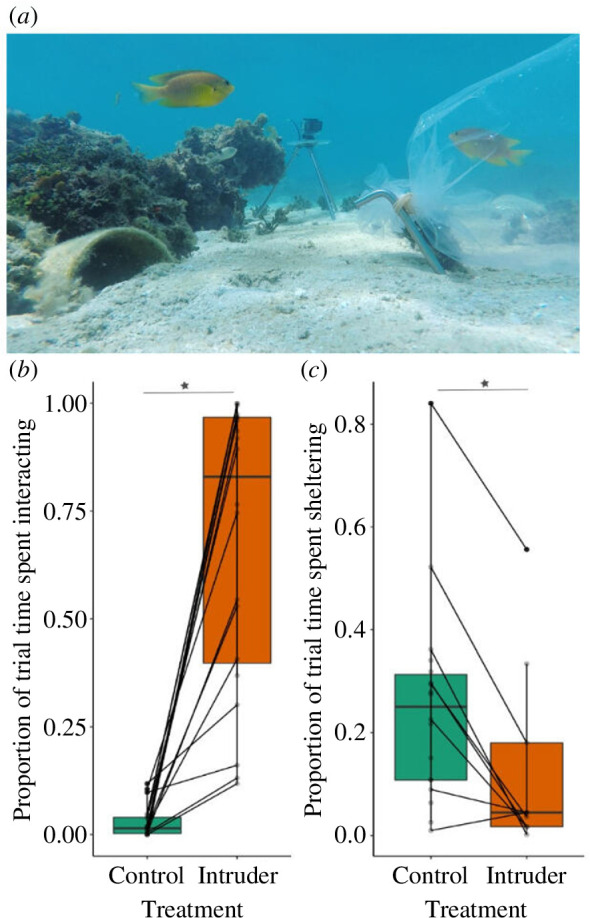
(***a***) Intruder treatment in Experiment 1 with a conspecific contained within a transparent bag near the focal nest. Boxplots of the proportion of trial time spent by nest-defending male Ambon damselfish (***b***) interacting with a transparent bag and (***c***) sheltering when the bag contained either a conspecific intruder or just seawater (control). Horizontal black lines represent the median value per treatment and coloured boxes show an interquartile range of treatment responses. Vertical lines represent the boxplot whiskers showing the maximum and minimum of the treatment responses. Points represent raw data values, with diagonal lines connecting the paired data from individuals across the two treatments. *p < 0.05. *n* = 20 males who received both treatments.

**Figure 2.**
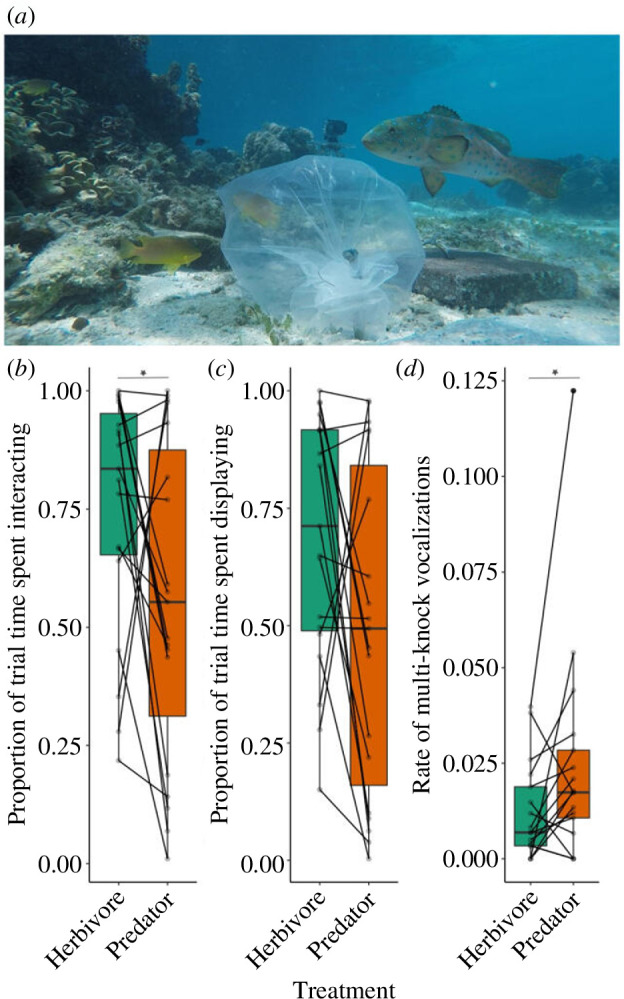
(***a***) Predator treatment in Experiment 2 with a conspecific intruder in a transparent bag and a predator model presented near the focal nest. Boxplots showing the proportion of trial time spent by nest-defending male Ambon damselfish (***b***) interacting with and (***c***) visually displaying to a conspecific intruder, and (***d***) the rate (per second) of multi-knock vocalizations produced, when exposed to a predator model versus a herbivore model. Horizontal black lines represent the median value per treatment and coloured boxes show the interquartile range of the treatment responses. Vertical lines represent the boxplot whiskers showing the maximum and minimum of the treatment responses. Points indicate raw data values with diagonal lines connecting the paired data from individuals across the two treatments. *p < 0.05. (***a***) *n* = 19, (***b***) *n* = 19 and (***c***) *n* = 17 males who received both treatments.

For each trial in Experiment 1, we attached an 8 l transparent plastic bag—containing a conspecific in seawater (intruder treatment; figure 1*a*) or just seawater (control treatment)—to a metal stake 0.5 m from the focal nest entrance (as per [23,24]). In both treatments in Experiment 2, we placed a bagged conspecific intruder near the focal nest entrance as in Experiment 1. For each trial in Experiment 2, we also presented one of three model exemplars of either a predatory coral grouper (*Plectropomus leopardus*) (figure 2*a*) or a herbivorous brown surgeonfish (*Acanthurus nigrofuscus*) ~1 m from the nest entrance, at the same time as the intruder; see electronic supplementary material (including figure S1) for further details of models and their placement. In both experiments, bag placement triggered a 15-min behavioural-response recording period; as males often immediately react to the presence of an intruder, there was no acclimation period.

From the GoPro footage of each trial, we coded behaviour (see electronic supplementary material, table S1) using Behavioural Observation Research Interactive Software (BORIS) version 8.0.9 [25]. We scored time spent by the focal male sheltering (in the nest or adjacent rubble) and interacting with the intruder (within two body lengths of it); when it was interacting, we scored the number of aggressive acts (chasing, striking and darting) directed at the intruder (as per [23,24]). Time when the focal fish was not in view was also recorded. From a signalling perspective, we scored the time visually displaying (extending the anal and dorsal fin and/or fanning the tail towards the intruder) and counted occurrences of single or multiple pulses (vocal ‘syllables’ within 1 s of each other) of aggressive ‘wipe’ and ‘knock’ vocalizations [22,26–29]. These vocalizations are acoustically distinct in recordings and when visualized in spectrograms (electronic supplementary material, figure S2). Video scoring of Experiment 1 was done blind to treatment. This was not possible for Experiment 2 as the model type was sometimes visible, but scoring was completed by a naive observer (J.E.S.). In addition, a subset of videos was re-watched and scored by a second observer (I.K.D.) to ensure that there was high inter-rater reliability (see electronic supplementary material for further details).

We carried out all statistical analyses in RStudio version 1.4.1103 [30] using proportions of time and event rates as dependent variables (see electronic supplementary material for further details). Since the assumptions of parametric testing were not met, Wilcoxon signed-rank tests were used to compare responses in the two treatments of an experiment. Owing to occasional camera-recording failures, total sample sizes for analyses were 20 paired trials in Experiment 1 and 19 paired trials in Experiment 2. Proportions of time spent sheltering, interacting and visually displaying were calculated from the time that the focal fish was visible. Rates of aggressive acts, as well as single and multiple pulse vocalizations (considered separately to assess the intensity of vocalizations), were calculated from the time that the focal male spent interacting with the intruder.

## 3. Results

In Experiment 1, nest-defending males spent a greater proportion of time interacting with a conspecific intruder than an empty bag (Wilcoxon signed-rank test: *V* = 210, *n* = 20, *p* < 0.001; figure 1*b*). This was, at least in part, because males spent less time sheltering when there was a conspecific present near their nest (*V* = 165, *n* = 20, *p* < 0.001; figure 1*c*); however, even in the time outside their shelter (i.e. in open water), males still spent a greater proportion of time interacting with an intruder compared to an empty bag (*V* = 210, *n* = 20, *p* < 0.001). Nest-defending males only spent time visually displaying (mean ± s.d. proportion of time: 0.57 ± 0.41, range = 0–1) and conducting aggressive acts (mean ± s.d. rate: 0.04 ± 0.06 acts per second, range = 0–0.64) when faced with an intruder; neither behaviour was displayed in the control treatment. There was no acoustic signalling when exposed to just an empty bag; production of both wipe (mean ± s.d. rate, single pulse: 0.1 ± 0.2 acts per second, range = 0–0.9; multiple pulse: 0.1 ± 0.1 acts per second, range = 0–0.4) and knock (single pulse: 0.1 ± 0.03 acts per second, range = 0–0.1; multiple pulse: 0.04 ± 0.04 acts per second, range = 0–0.1) vocalizations only occurred when there was a conspecific intruder nearby.

In Experiment 2, nest-defending males spent a smaller proportion of time interacting with the conspecific intruder when there was a predator model compared to a herbivore model (Wilcoxon signed-ranks test: *V* = 46, *n* = 19, *p* = 0.049; figure 2*b*). Even when outside their shelter, there was a non-significant tendency for males to interact less with a conspecific intruder when a predator model compared with a herbivore model was nearby (*V* = 42, *n* = 19, *p* = 0.061). There was no significant treatment difference in the time that males spent sheltering (*V* = 68, *n* = 19, *p* = 0.294) or in their rate of aggression towards the intruder (*V* = 88, *n* = 18, *p* = 0.932). There was a non-significant trend for males to spend less time visually displaying to the intruder when there was a predator rather than a herbivore model (*V* = 48, *n* = 19, *p* = 0.060; figure 2*c*). Once interacting with the intruder, however, there was no significant difference in the proportion of time spent visually displaying between herbivore and predator model treatments (*V* = 55, *n* = 19, *p* = 0.113). In terms of acoustic signalling, there were no significant treatment differences in the rate of single pulse (*V* = 28, *n* = 13, *p* = 0.244) or multi-pulse (*V* = 14, *n* = 11, *p* = 0.102) wipe vocalizations nor in single pulse knock vocalizations (*V* = 71, *n* = 17, *p* = 0.818). However, nest-defending males did exhibit a significantly greater rate of multi-pulse knock vocalizations when there was a predator model compared with a herbivore model (*V* = 33, *n* = 17, *p* = 0.040; figure 2*d*).

## 4. Discussion

We found that nest-defending male Ambon damselfish responded aggressively to a conspecific intruder, spending less time sheltering and more time interacting, as well as signalling both visually and acoustically. There was some evidence that behavioural responses were modified in the simulated presence of a predator: males spent less time interacting with the intruder, with a tendency towards reduced investment in visual displays and an increase in certain forms of acoustic signalling instead.

Aggressive nest defence not only reduces time and energy to invest in other activities such as foraging or vigilance but also renders an individual more vulnerable to predation. The decreased interaction with a conspecific intruder by male damselfish in the presence of a predator model may therefore reflect a trade-off between nest defence and minimizing predation risk. Such a trade-off is likely impacted by the type of predator, as seen in terrestrial species that alter their anti-predator response intensity depending on the threat level [31,32]. Coral groupers (the predator species that we modelled) hunt opportunistically, striking when prey are exposed or distracted [33]. Ambon damselfish might frequently be in the presence of a resident grouper without it posing a direct threat, and might therefore invest more in continued vigilance without halting other behaviours such as feeding or nest defence [34,35]. Other damselfish predators, such as jacks (Carangidae) or barracudas (Sphyraenidae), are more transient and attack from open water through fast chases [21,36]. In the presence of these predators, a damselfish might trade-off nest defence and anti-predator behaviour entirely, choosing to shelter immediately [21,34], but future work would be needed to test responses to different predator types.

Male Ambon damselfish interacted less with a conspecific intruder (i.e. spent less time close to them) in the presence of a predator. Consequently, the overall proportion of time spent visually displaying tended to be lower; the proportion of time visually displaying when interacting with the intruder did not significantly differ in the presence of a predator compared to a herbivore model. Males did, however, exhibit a concomitant increase in the production of multi-pulse knock vocalizations, highlighting the potentially flexible use of multimodal signalling. Knock vocalizations are associated with nest defence and aggression [22,29]; multiple pulses, or pulse trains, have been linked with escalated aggressive displays in fish [37]. Acoustic signals can relay information about the signaller [38–40], informing an intruder about their condition, dominance and willingness to fight without necessitating defenders to approach the intruder and any potential predators in the process. Vocalizations, therefore, offer an effective form of nest-defence signalling under predation risk. Vocal fish often respond acoustically to predators [41] and can use acoustic signals for dual purposes [10]. The multi-pulse knock vocalizations of the Ambon damselfish, which increased in the presence of a conspecific intruder and predator, could potentially deter the predator as well as the conspecific intruder. Multimodal signals can contain independent components aimed at different receivers, even for differing purposes [42,43], but a more nuanced understanding of both their complexity and context-specific information requires further exploration, especially in the wild.

Behavioural flexibility is important when animals take into account, for instance, past interactions with conspecifics [44], predator risk [45] and environmental alterations [46]. Our experiments were conducted when there were no eggs in the nest; it is possible that there could be even stronger defence against conspecific intruders and/or a different trade-off with anti-predator behaviour when there are eggs present. In general, individuals who can appropriately respond to changing circumstances, trading-off their risk tolerance with other needs, will likely have a better chance of surviving and reproducing [47]; multimodal signalling can provide inherent plasticity to these trade-offs [12,15,43]. Our study is, to the best of our knowledge, the first that demonstrates experimentally in the wild how multimodal signalling in coral reef fish can mediate the trade-off between defensive and anti-predator responses. This capacity is especially important now given the rate at which the world in general, and coral reefs in particular, are changing owing to anthropogenic disturbances.

## Data Availability

The datasets and R code used for analysis have been submitted to Dryad and Zenodo respectively under 'Stage 1 & 2 total duration and count in behaviour trials_Context-dependent multimodal behaviour in a coral reef fish' [48]. Electronic supplementary material is available online [49].
